# About-face on the metabolic side effects of rapamycin

**DOI:** 10.18632/oncotarget.3354

**Published:** 2015-02-14

**Authors:** Adam B. Salmon

**Affiliations:** The Sam and Ann Barshop Institute for Longevity and Aging Studies and Department of Molecular Medicine, The University of Texas Health Science Center at San Antonio and Geriatric Research, Education and Clinical Center, South Texas Veterans Health Care System, San Antonio, TX, USA

Rapamycin, an inhibitor of the mTOR pathway, can extend lifespan and improve age-related functional decline in mice, thereby providing the first proof of principal that a pharmaceutical agent can slow the aging process in mammals [1, 2]. These outcomes have proven robust in repeated studies, however, their potential translational relevance towards a means to slow aging or prevent age-related disease in otherwise healthy humans remains unclear. Part of the challenge in addressing the potential of rapamycin (or its analogs) as a pro-longevity therapeutic lies in its known clinical risks for adverse side-effects. Primary amongst these are metabolic defects that include hyperglycemia, hyperlipidemia, insulin resistance and increased incidence of new-onset type 2 diabetes. In healthy rodents, treatment with rapamycin also causes a relatively rapid, dose-dependent impairment of markers of glucose homeostasis [2]. The nature of the metabolic effects/defects caused by rapamycin remain ambiguous in regards to their role in longevity and healthy aging. Fang et al. suggested the effects of rapamycin on metabolism depend on the length of treatment with a detrimental effect on glucose metabolism in the short-term whereas mice treated chronically with rapamycin actually became insulin-sensitive [3]. On the other hand, Blagosklonny has proposed that the presumed metabolic impairments caused by rapamycin may simply be a consequence of its action as a “starvation-mimetic” and, further, may be fundamentally required for its pro-longevity effect [4].

Clarifying these uncertain relationships could pave the way to understanding how rapamycin, and thus targeting mTOR, could be used in ways that maximize its benefit during treatment. To this goal, several significant questions may be raised, the first of which is the molecular nature of the metabolic impairments imparted by rapamycin. Several recent studies have elegantly shown that chronic treatment with rapamycin inhibits mTORC2 signaling which may be a primary culprit in its alteration of glucose metabolism. Interestingly, Lamming et al. show that metabolic effects of reduced mTORC2 are independent of those on lifespan in mice with deletion of *Rictor* [5]. Because rapamycin has now been shown to be promiscuous in its inhibition of the mTOR complexes, approaches that specifically target mTORC1 may help in this regard.

A second question that has arisen is whether the metabolic impairments caused by rapamycin can be alleviated. These effects in mice are dose-dependent [2]

though until recently it has been unclear whether such treatment with rapamycin causes a permanent alteration in metabolic function. To better understand this question, we designed a straightforward study, now published in *Aging*, that tested whether the metabolic deficiencies that occur in mice chronically treated with encapsulated rapamycin are retained after the cessation of treatment [6]. We also took this opportunity to test whether diet, genotype, and time of treatment could alter the metabolic effects of rapamycin treatment. In this study, glucose homeostasis, as measured by glucose and insulin tolerance tests, was significantly impaired by rapamycin in both inbred (C57BL/6) and genetically heterogeneous (UT-HET3) mice. This rapamycin-induced impairment occurred in mice fed either low fat or high fat diets and, moreover, we found no evidence that increasing the length of treatment promoted any improvement in metabolic phenotypes as has previously been suggested [3]. However, once we ended the rapamycin treatment, all markers of glucose homeostasis almost completely returned to normal; i.e., glucose homeostasis in previously rapamycin-treated mice was no different from those never treated with rapamycin. Perhaps most interestingly, this reversal occurred in a relatively short period of time (1-2 weeks) even after long term (4 months) rapamycin treatment, suggesting that these metabolic side effects are a consequence of ongoing use of this drug and are not permanent alterations to the glucose homeostatic network.

Many of the pre-translational concerns regarding the usefulness of targeting the mTOR pathway revolve around discovering ways to mitigate its side effects while still retaining the beneficial effects. Our finding that the metabolic impairments caused by rapamycin can be alleviated suggest that alternative treatment regimens might be a feasible step towards this goal. One possibility could be intermittent treatment with rapamycin; Leontieva *et al* recently showed that a once-weekly treatment with rapamycin extends lifespan in high fat-fed mice without altering glucose or insulin levels [7]. A second possibility might be pairing rapamycin with therapeutic treatment for metabolic dysfunction. Rosiglitazone, an insulin sensitizer, can partially improve the glucose impairments caused by rapamycin when administered concurrently [8]. To test the effects on lifespan of such an approach, the NIA's Intervention Testing Program is currently performing longevity studies in which mice are treated concurrently with rapamycin and the antidiabetic drug metformin.

These findings will be an important piece in solving the puzzle regarding the complicated role of rapamycin (and mTOR) in metabolism and longevity.
